# Supplementary material for: Genome-Scale Mining of Acetogens of the Genus Clostridium Unveils Distinctive Traits in [FeFe]- and [NiFe]-Hydrogenase Content and Maturation
Source: Microbiol Spectr. 2022 Jun 23;10(4):e01019-22. doi: 10.1128/spectrum.01019-22 (PMC9431212; doi:10.1128/spectrum.01019-22)
Supplement: Supplemental file 1 — Supplemental material. Download spectrum.01019-22-s0001.pdf, PDF file, 2.8 MB [file spectrum.01019-22-s0001.pdf]

Genome-scale mining of acetogens of the genus *Clostridium* unveils distinctive traits in [FeFe]- and [NiFe]-hydrogenase content and maturation

Supplementary Information

Supplementary Materials and Methods

Acetogen genomic dataset

Deatiled information about the genomes of the clostridial acetogens retrieved from the NCBI Assembly database is listed below:

- **Clostridium autoethanogenum DSM 10061:** - assembly: ASM148472v1; RefSeq Sequence (version): NZ_CP012395; 26-04-2020; 4352466 bp; Genes (total) 4074; CDSs (total) 3973; Genes (coding) 3848; CDSs (with protein) 3848; Genes (RNA) 101; rRNAs 9, 9, 9 (5S, 16S, 23S); complete rRNAs 9, 9, 9 (5S, 16S, 23S); Pseudo Genes (total) 125;
- **Clostridium ljungdahlii DSM 13528** - assembly: ASM14368v1; RefSeq Sequence (version): NC_014328.1; 27-10-2020; 4630065 bp; Genes (total) 4309; CDSs (total) 4203; Genes (coding) 4124; CDSs (with protein) 4124; Genes (RNA) 106; rRNAs 9, 9, 9 (5S, 16S, 23S); complete rRNAs 9, 9, 9 (5S, 16S, 23S); Pseudo Genes (total) 79;
- **Clostridium carboxidivorans P7** - assembly: ASM103862v1; RefSeq Sequence (version) Chromosome: NZ_CP011803.1; 16-07-2020; 5732880 bp; Genes (total) 5,272; CDSs (total) 5158; Genes (coding) 5098; CDSs (with protein) 5098; Genes (RNA) 114; rRNAs 11, 11, 11 (5S, 16S, 23S); complete rRNAs 11, 11, 11 (5S, 16S,

23S); Pseudo Genes (total) 60. RefSeq Sequence (version) Plasmid: NZ_CP011804.1; 16-07-2020; 19902 bp;

- **Clostridium drakei SL1** - assembly: ASM309617v1; RefSeq Sequence (version): NZ_CP020953.1; 17-02-2020; 5695241 bp; Genes (total) 5,154; CDSs (total) 5,027; Genes (coding) 4,901; CDSs (with protein) 4,901; Genes (RNA) 127; rRNAs 10, 10, 10 (5S, 16S, 23S); complete rRNAs 10, 10, 10 (5S, 16S, 23S); Pseudo Genes (total) 126;
- **Clostridium scatologenes ATCC 25775** - assembly: ASM96837v1; RefSeq Sequence (version): NZ_CP009933.1; 03-11-2020; 5749410 bp; Genes (total) 5,272; CDSs (total) 5,176; Genes (coding) 5,046; CDSs (with protein) 5,046; Genes (RNA) 96; rRNAs 9, 9, 9 (5S, 16S, 23S); complete rRNAs 9, 9, 9 (5S, 16S, 23S); Pseudo Genes (total) 130;
- **Clostridium sp. AWRP** - assembly: ASM400639v2; RefSeq Sequence (version): NZ_CP029758.2; 20-06-2020; 4579117 bp; Genes (total) 4,324; CDSs (total) 4,220; Genes (coding) 4,105; CDSs (with protein) 4,105; Genes (RNA) 104; rRNAs 9, 9, 9 (5S, 16S, 23S); complete rRNAs 9, 9, 9 (5S, 16S, 23S); Pseudo Genes (total) 115;
- **Clostridium ragsdalei P11** - assembly: ASM167516v1; 22-06-2016; 4424992 bp;
- **Clostridium coskatii PTA-10522** - assembly: ASM167520v1; 22-06-2016; 4538837 bp.

Construction of hydrogenase gene tree

We conducted the evolutionary analysis in MEGA X (1). After aligning the amino acid sequences of the validated hydrogenase catalytic subunits by using ClustalW algorithm (2), the evolutionary tree was inferred by using the Maximum Likelihood method

and JTT matrix-based model (3). We used default parameters, except for the number of bootstrap replications that was set to 250. The percentage of trees in which the associated protein sequences clustered together is shown next to the branches. Initial trees for the heuristic search were obtained automatically by applying Neighbor-Join and BioNJ algorithms to the matrix of pairwise distances estimated using the JTT model, and then selecting the topology with superior log likelihood value. The tree is drawn to scale, with branch lengths measured in the number of substitutions per site.

Detection of putative genes encoding hydrogenase maturation proteins

Since the framework of maturation pathway differ between [FeFe]- and [NiFe]-hydrogenases, we separately outline the procedures adopted to identify putative maturation protein encoding genes in the genomes of clostridial acetogens.

[FeFe]-hydrogenase maturation proteins

To verify the presence of [FeFe]-hydrogenase maturation genes, we performed a sequence-based alignment (blastp with standard parameters) of known HydE, HydF, and HydG proteins against the amino acid sequences of each acetogen. As queries in the alignment-based analysis, we used the amino acid sequences of the three maturation genes whose crystallographic structures are available in the Protein Data Bank (PDB) database: HydE (PDB ID: 3CIW) and HydF (PDB ID: 5LAD) were retrieved from *Thermotoga maritima*, and HydG (PDB ID: 4WCX) was retrieved from *Thermoanaerobacter italicus*. Regardless of the number of hits obtained for each query sequence, we inspected each hit searching for the conserved amino acids signatures reported by the literature to date for the recognition of maturation proteins.

For HydE and HydG, known to be radical *S*-adenosylmethionine (SAM) enzymes, we initially searched the protein sequences of the alignment hits for the conserved N-terminal radical SAM motifs C-x(3)-C-x(2)-C (4, 5, 6, 7) and C-x(2)-C-x(4)-C (8). The amino acid sequences of the hits were then inspected to identify the presence of features related to the C-terminal region of the radical SAM proteins. In particular, for HydE we searched for the presence of the C-x(7)-C-x(2)-C motif responsible for the coordination of an optional Fe-S cluster (5, 9, 10) and the conserved Y-x(2)-Y motif, which could play a role in either catalysis or structure stability (10). For HydG, we assessed the presence of the C-x(2)-C-x(22)-C and C-x(5)-C-x(19)-C [7] motifs, responsible for coordination of a Fe-S cluster, which is thought to have an essential catalytic role (4, 6, 7, 11, 12).

HydF, which belongs to the GTPase protein family, shares with them a homologous N-terminal domain, which contains crucial conserved amino acid motifs indispensable for a successful hydrogenase maturation. Thus, we looked for the following motifs: Walker A P-loop motif, G-x(4)-G-K-[S/T]; Walker B Mg^{2+} binding motif, h(4)-D-x-(2)-G (h, hydrophobic); GTP-specific distal motif, [N/T]-K-x-D (4, 13, 14). Moreover, we searched for a feature related to C-terminal region of HydF proteins, the conserved C-x-H-x(46-53)-C-x(2)-C motif responsible for coordination of an Fe-S cluster (4, 14, 15, 16). For all of the identified maturation proteins, we checked if their amino acidic sequence length agrees with that reported in the literature.

[NiFe]-hydrogenase maturation proteins

To verify the presence of the [NiFe]-hydrogenase maturation genes in the genome of the clostridial acetogens, we performed a sequence-based alignment of the six maturation proteins, required in the maturation process, against the amino acid sequences of each acetogen. As queries for the alignment analysis, we used the amino acid sequences corresponding to the six maturation genes (*hypABCDEF*) from *Thermococcus*

kodakarensis, of which the crystallographic structures are also available in PDB with the following codes: 3A43; 3VX3; 2Z1C; 2Z1D; 2Z1F; 4G91. For HypB, due to the both reported ATP-ase (*T. kodakarensis)* and GTP-ase type, we also used the amino acid sequence corresponding to HypB from *Helicobacter pylori* (PDB ID: 4LPS), which is known to use GTP. Similar to the procedure used for [FeFe]-hydrogenase maturation proteins, we assessed the presence of amino acid sequence motifs known to support the functionality of the maturation proteins. In the case of HypA, we searched for two signatures: at the N-terminal, the conserved amino acids M-H-E for the Nickel binding (17), and, at the C-terminal, the conserved Zinc binding motif, C-x(2)-C-x(n)-C-P-x-C with n=12 (18, 19). For HypB, we searched for the conserved C-H-x(n)-C motif, with the Cys and the Histidine (His) residues responsible for the binding of the metal, and close to that motif, we assessed the presence of a strongly conserved aromatic residue. Furthermore, we checked the presence of the nucleotide binding motif [N/S]-K-x-D (19, 20). For HypC, we searched for two conserved catalytically important residues, Cys and His (Cys2 and His54 in *T. kodakarensis*) (19, 21). For HypD, we searched for the four reported conserved motifs C-G-x-H, G-P-G-C-P-V-C, G-F-E-T-T, and P-x-H-V-S, and for the typical conserved [4Fe-4S] cluster binding motif, C-x(14)-C-x(6)-C-x(16)-C (18, 19). For HypE, we searched for the conserved C-terminal motif P-R-[I/V]-C (19). For HypF, we searched for the presence of the conserved zinc binding motif C-x(2)-C-x(18)-C-x(2)-C (19). For all of the identified maturation proteins, we checked if the amino acidic length of each one is also in agreement with that reported in the literature.

Diaphorase subunits of group A4 and group A3 [FeFe]-hydrogenases

We compared the sequences and the structural features of putative cofactor binding subunits with the NuoF subunit of *T. thermophilus* (PDB ID: 6ZIY). By using the MUSCLE algorithm [22], we built a multiple sequence alignment of NuoF from *T. thermophilus* with

the putative subunits harbouring the NuoF domain, to check if the motifs responsible for the interaction with the cofactor in NuoF are present in the putative cofactor binding subunits identified in the hydrogenases. We then compared the secondary structure context of the putative cofactor binding subunits with that of NuoF from *T. thermophilus*. To this aim, we predicted the secondary structure of the putative cofactor binding subunits by gathering the “Predicted Secondary Structure” output of the I-TASSER server (23, 24, 25) and we superimposed the predicted secondary structure elements to the multiple sequence alignments. Finally, we checked if the diaphorase models generated by I-TASSER suggest the presence of a cavity similar to the cavity that accommodates FMN and NADH in NuoF. For each subunit, we used the I-TASSER-derived model with the highest C-score.

Subcellular localization analysis

We analysed the protein sequences corresponding to the small subunits of the predictyed [NiFe]-hydrogenase belonging to the group 1a by Psortb v3.0.3 (26). We scanned the small subunit protein sequences by the ScanProsite tool (27) searching for the presence of instances of the R-R-x-F-x-K motif that is commonly recognized by the Tat pathway. Finally, we searched the sequences for Tat signal peptides using TatP (28), for Gram-positive bacteria.

Search for cytochrome biosynthetic genes in the genomes of acetogens of the genus *Clostridium*

Prokaryotes accomplish cytochrome c biosynthesis by the System I (29, 30) or System II (31) pathway. System I is comprised of up to ten proteins (CcmABCDEFGHI, CcdA (cytochrome c defective) or DsbD (disulfide bond formation protein)), whereas

System II of minimally two proteins (CcsBA) that are dedicated strictly for cytochrome *c* biogenesis. The coproporphyrin-dependent (CPD) heme biosynthesis pathway consisting of five genes accomplishes thecytochrome b biosynthesis (32). To assess the presence of putative genes involved in such pathways, we run a sequence similarity search by blastp with default parameters. We used the following protein sequences: *Chlamydomonas Reinhardtii* CcsA and *Chlamydomonas Reinhardtii* CcsB of System II, the amino acid sequences of the Ccm present in the purple nonsulfur bacterium *Rhodobacter capsulatus or* in *Escherichuia coli*, and the protein sequences of the CPD pathway genes in *Escherichia coli.*

Supplementary Results

Subcellular localization of [NiFe]-hydrogenases in clostridial acetogens

The small subunits of H_2-uptake [NiFe]-hydrogenases usually possess a large signal peptide containing a conserved R-R-x-F-x-K motif, which serves as signal recognition to target fully folded mature uptake hydrogenases to the membrane thanks to the recognition by a specific protein translocation pathway designated the membrane targeting and translocation (Mtt) (33) or twin arginine translocation (Tat) pathway (34, 35). An attempt to predict the subcellular localization of the identified [NiFe]-hydrogenase subunits via PSORTb v3.0.3 (26) suggested a cytoplasmic membrane localization, based on sequence similarity to the quinone-reactive [NiFe]-hydrogenase small chain HydA in *Wolinella succinogenes* DSM 1740 (36), as shown in **Supplementary Table 2**. However, the expected RR-containing motif was not recognizable in the predicted small subunits of the [NiFe]-hydrogenases. Similarly, no evidence for twin-arginine signal peptides was gathered in any of the predicted [NiFe]-hydrogenase small subunit when we analysed the protein sequences by TatP (28), which integrates pattern matching and machine learning

for coping with the Tat signal peptide prediction problem. It is possible that the [NiFe]-hydrogenase small subunits could carry unusual signal pepides since naturally occurring Tat signal peptide variants have been previously reported mainly for *E. coli* (37, 38, 39, 40). Alternatively, we cannot rule out the possibility that these hydrogenases resort to the general Sec protein transport (41, 42) or that their subcellular localization is cytoplasmic, although the H_2-uptake [NiFe]-hydrogenases are expected to be periplasmic.

Hydrogenase content in *C. ljungdahlii*

The survey of the *C. ljungdahlii* genome indicates that it encodes one dimeric group 1a [NiFe]-hydrogenase (catalytic subunit CLJU_RS14140), one monomeric group B [FeFe]-hydrogenase (catalytic subunit CLJU_RS09955), two group A4 [FeFe]-hydrogenases – of which one hexameric (catalytic subunit CLJU_RS03480) and one trimeric (catalytic subunit CLJU_RS08485) -, and one trimeric group A3 [FeFe]-hydrogenase (catalytic subunit CLJU_RS07205), as shown in **Figure 3**.

The monomeric group B [FeFe]-hydrogenase CLJU_RS09955 corresponds to CLJU_c20290 according to (43).

The group 1a [NiFe]-hydrogenase identified consists of the large subunit, encoded by CLJU_RS14140, and the small subunit, encoded by CLJU_RS14145. In full similarity to CLAU_RS04060-RS04065, we could not identify the accessory cytochrome subunit, which is a common trait of group 1a [NiFe]-hydrogenases and which was verified in the best hit (WP_010890826.1) of the catalytic subunit CLJU_RS14140 in the controlled repository HydDB. Noteworthily, the authors in ref. [9] reported this enzyme as dimeric hydrogenase encoded by CLJU_c28660-c28670.

The gene cluster CLJU_RS03460-RS03485 (corresponding to CLJU_c07030-c07080) encodes the group 4 [FeFe]-hydrogenase known as the electron bifurcating NADP- and ferredoxin-dependent hydrogenase HytABCDE$_1$E$_2$. CLJU_RS03480 encodes the catalytic subunit HytA while CLJU_RS03465 encodes the iron-sulfur flavoprotein HytB, which was predicted to harbour the site binding the redox cofactor. CLJU_RS03460, CLJU_RS03470, CLJU_RS03475, and CLJU_RS03485 encode, respectively, the Fe-S subunits HytC, HytD, HytE1 and HytE2. An additional group A4 [FeFe]-hydrogenase was predicted to be encoded by CLJU_RS08480-RS08490 with CLJU_RS08485 acting as the catalytic subunit. Similarily to CLAU_RS18770-RS18765 in *C. autoethanogenum*, we counted CLJU_RS08480-RS08490 among the predicted hydrogenases even though we could not annotate the third predicted subunit CLJU_ RS08490 as a Fe-S protein, which is expected according to the genetic organization of the best hit (WP_013238388.1) of the catalytic subunit in the sequence similarity analysis against HydDB.

According to our analysis, the genes CLJU_RS07205-RS07215 encode a group A3 [FeFe]-hydrogenase which is predicted to operate in electron bifurcation mode. Indeed, besides CLJU_RS07205, which encodes the catalytic subunit, we predicted CLJU_RS07210 as the redox cofactor binding subunit. According to the genetic organization of this hydrogenase subgroup, we expected a third subunit was expectable that we identified in the thioredoxin CLJU_RS07215. The genes encoding this hydrogenase were identified as CLJU_c14700-c14720 in ref. (43). In a similar way to that observed at the transcript level in *C autoethanogenum*, HytABCDE$_1$E$_2$ was found the most highly expressed hydrogenase followed by the [FeFe]-hydrogeases CLJU_RS08480-RS08490, CLJU_RS09955 whereas the group 1a [NiFe]-hydrogenase CLJU_RS14140-RS14145 was barely detectable in *C. ljungdahlii* grown in H_2/CO_2 (44), as shown in **Supplementary Table 5**.

Hydrogenase content in *C. coskatii*

C. coskatii features the same hydrogenase repertoire as *C. autoethanogenum* and *C. ljungdahlii* encoding one dimeric group 1a [NiFe]-hydrogenase, one monomeric group B [FeFe]-hydrogenase (CLCOS_RS01500), two group A4 [FeFe]-hydrogenases, and one trimeric group A3 [FeFe]-hydrogenase, as shown in **Figure 4**. The group 1a [NiFe]-hydrogenase consists of the large (CLCOS_RS05460) and small subunits (CLCOS_RS05465) and lacks the expectable third cytochrome subunit. Unlike the group A4 [FeFe]-hydrogenases in *C. autoethanogenum* and *C. ljungdahlii*, we were able to confirm that the genetic organization of this enzyme agrees with the template reported in HydDB since the gene encoding the catalytic subunit (CLCOS_RS06485) was found to be flanked by two annotated Fe-S proteins, CLCOS_RS06480 and CLCOS_RS06490. The trimeric group A3 [FeFe]-hydrogenase consists of the catalytic subunit CLCOS_RS00120, the diaphorase CLCOS_RS00125 and the thioredoxin CLCOS_RS00130. The hexameric group A4 [FeFe]-hydrogenase was predicted to be encoded by CLCOS_RS18170-RS18195 with CLCOS_RS18190 acting as the catalytic subunit. The Fe-S flavoprotein CLCOS_RS18175 is ortholog of CLAU_RS13685 and CLJU_RS03465 in *C. autoethanogenum* and *C. ljungdahlii*, CLCOS_RS18170, CLCOS_RS18180, CLCOS_RS18185, and CLCOS_RS18195 encode, respectively, the Fe-S subunits HytC, HytD, $HytE_1$ and $HytE_2$. In agreement with the formate dehydrogenase-linked function attributed to this type of hydrogenases, it is noteworthy that the hydrogenase encoding genes CLCOS_RS18170-RS18195 co-localize with genes encoding a putative formate dehydrogenase (CLCOS_RS18150-RS18165).

Hydrogenase content in *C. sp. AWRP*

The genome of *C.* sp. AWRP features the same hydrogenase repertoire as *C. autoethanogenum* and *C. ljungdahlii* and *C. coskatii* encoding one dimeric Group 1a [NiFe]-hydrogenase, one monomeric Group B [FeFe]-hydrogenase, two Group A4 [FeFe]-hydrogenases, and one trimeric Group A3 [FeFe]-hydrogenase, as shown in **Figure 4**. The hydrogenases identified in this study were corroborated by referring to ref. (45), which describes the isolation and sequencing of the genome of this acetogenic strain. With the exception of DMR38_18550, we confirmed the same set of hydrogenases reported in ref. (45), even though the authors did not provide any functional or structural characterization. DMR38_18550 was included among the group B [FeFe]-hydrogenases thatwe identified purely based on sequence similarity to the controlled hydrogenases' collection stored in HydDB. However, it was finally discarded owing to the apparent lack of the sequence patterns, which are known to surround the cysteine ligands of the catalytic metal sites of [FeFe]-hydrogenases. On the other hand, we identified the monomeric group B [FeFe]-hydrogenase DMR38_RS10360 that corresponds to DMR38_10370 in ref. (45) . The group 1a [NiFe]-hydrogenase consists of the large (DMR38_RS14545) and small subunits (DMR38_RS14550) and lacks the expectable third cytochrome subunit, similarily to the corresponding hydrogenases in *C. autoethanogenum*, *C. ljungdahlii*, and *C. coskatii*. Accordingly, (45) reported this hydrogenase as a dimeric [NiFe]-hydrogeanse (DMR38_14570-14575). We also detected two trimeric [FeFe]-hydrogenases. DRM38_RS07425-RS07435 is a trimeric Group A3 [FeFe]-hydrogenase whose catalytic subunit was predicted to be encoded by DMR38_RS07425. In agreement with the electron bifurcation operational mode attributed to this hydrogenase, we identified DMR38_RS07430 as the putative redox cofactor binding subunit. This hydrogenase was also reported by ref. (45) as DMR38_07425-07435. DRM38_RS08575-RS08585 is the other trimeric hydrogenase that belongs to Group A4 [FeFe]-hydrogenases. As previously shown for the trimeric Group A4 [FeFe]-hydrogenase in *C. coskatii*, the reconstruction of

the genetic organization of this hydrogenase is in agreement with the template reported by HydDB for this subgroup, and is composed by the catalytic subunit DRM38_RS08580 and by two Fe-S cluster-containing subunits, DMR38_RS08575 and DMR38_RS08585. This hydrogenase corresponds to DMR38_08575-08585 in ref. (45). The hexameric Group A4 [FeFe]-hydrogenase is encoded by DMR38_RS03365-RS03390 corresponding to DRM38_03365-03390 in ref. (45). The gene DMR38_RS03385 encodes the catalytoc subunit and the gene DMR38_RS03370 is an iron-sulfur flavoprotein and may be the binding site for the redox cofactor. DRM38_RS03365, DRM38_RS03375, DRM38_RS03380, and DRM38_RS03390 encode, respectively, the Fe-S subunits HytC, HytD, $HytE_1$ and $HytE_2$. Moreover, in the same way as the other *Clostridium* acetogens studied, the hydrogenase genesco-localize with those of a putative formate dehydrogenase (DMR38_RS03345-RS03360).

Hydrogenase content in *C. ragsdalei*

The survey of the *C. ragsdalei* genome indicates that it encodes one dimeric group 1a [NiFe]-hydrogenase, one monomeric group B [FeFe]-hydrogenase (CLRAG_RS12855), two group A4 [FeFe]-hydrogenases, and one trimeric group A3 [FeFe]-hydrogenase (CLRAG_RS10115 acting as the catalytc subunit, CLRAG_RS10110 as the diaphorase, and CLRAG_RS10105 as the thioredoxin), as shown in **Figure 5**. The group 1a [NiFe]-hydrogenase consists of the large (CLRAG_RS17285) and small subunits (CLRAG_RS17290) and lacks the expectable third cytochrome subunit, similarily to the corresponding hydrogenases in *C. autoethanogenum*, *C. ljungdahlii*, and *C. coskatii* and *C.* sp. AWRP. CLRAG_RS14500-RS14510 was predicted to encode a trimeric group 4 [FeFe]-hydrogenase. Similarily to the corresponding hydrogenases in *C. coskatii* and *C.* sp. AWRP, the genetic organization of this hydrogenase reflects the template reported by HydDB for this subgroup, and consists of two Fe-S cluster-containing subunits,

CLRAG_RS14500 and CLRAG_RS14510, besides the catalytic subunit CLRAG_RS14505. The hexameric Group A4 [FeFe]-hydrogenase is encoded by CLRAG_RS09305-RS09330, with CLRAG_RS09325 encoding the catalytoc subunit and the gene CLRAG_RS09310 encoding the iron-sulfur flavoprotein. CLRAG_RS09305, CLRAG_RS09315, CLRAG_RS09320, and CLRAG_RS09330 encode, respectively, the Fe-S subunits HytC, HytD, $HytE_1$ and $HytE_2$. Moreover, in the same way as the other *Clostridium* acetogens studied, the hydrogenase genes co-localize with those of a putative formate dehydrogenase (CLRAG_RS09285-RS09300).

Hydrogenase content in *C. drakei*

The survey of the *C. drakei* genome shows the same hydrogenase enzyme composition as *C. scatologenes* hosting three group A3 [FeFe]-hydrogeanses, one group 1a [NiFe]-hydrogenase, one group B [FeFe]-hydrogenase and one group A4 [FeFe]-hydrogenase, as shown in **Figure 6**. The monomeric Group B [FeFe]-hydrogenase is encoded by B9W14_RS24170, which correspomds to B9W14_22055 mentioned in ref. (46). Similar to *C. carboxidivorans* and *C. scatologenes*, the group 1a [NiFe]-hydrogenase fulfils the genetic organization common for these hydrogenases, and is composed by the large (B9W14_RS11015), small (B9W14_RS11010) and cytochrome (B9W14_RS11005) subunits. We note that, according to ref. (46), this hydrogenase was deemed dimeric (B9W14_10930-10935) since the presence of the cytchrome subunit was overlooked. Alongside the *C. scatologenes* genome, *C. drakei* was inferred to contain three trimeric Group A3 [FeFe]-hydrogenases, namely B9W14_RS06375- RS06385, B9W14_RS14610-RS14620, and B9W14_RS20215-RS20225.The catalytic subunit B9W14_RS20215 of the latter trimeric hydrogenase was mentioned also in ref. (46) but therein was erroneously deemed monomeric (B9W14_20095). Finally, we predicted that B9W14_RS20180-RS20205 encode the hexameric group A4 FeFe-hydrogenase, with B9W14_RS20185

predicted to encode the catalytic subunit and B9W14_RS20200 the Fe-S flavoprotein able to bind the redox cofactor. B9W14_RS20180, B9W14_RS20190, B9W14_RS20195, and B9W14_RS20205 encode, respectively, the Fe-S subunits HytC, HytD, $HytE_1$ and $HytE_2$. Genomically, in line with the expectable formate dehydrogenase-linked role, the hydrogenase encoding genes are co-localized with the formate dehydrogenase encoding gene B9W14_RS20210. We note that ref. (46) mentioned two additional uncharacterized hydrogenase clusters, B9W14_14030–14045 and B9W14_11370–11395. The latter cluster, which corresponds to B9W14_RS11475-RS11450), emerged also in our alignment-based sequence similarity analysis whereby it was attributed to the Group 4f [NiFe]-hydrogenases. In agreement with the genetic organization foreseen for this hydrogenase subgroup, we predicted the large and small subunits to be encoded by B9W14_RS11470 and B9W14_RS11475, the two antiporter-like subunits to be encoded by B9W14_RS11460 and B9W14_RS11465, and the two transmembrane subunits to be encoed by B9W14_RS11455 and B9W14_RS11450. In spite of the plausible reconstruction of the structural genes of Group 4f [NiFe]-hydrogenases, we discarded this enzyme since we were unable to detect the sequence patterns surrounding the cysteine ligands of the catalytic metal site in the large subunit. According to the RNA-Seq analysis carried out in *C. Drakei* culture grown on H_2/CO_2 gas mixture, the most highly expressed hydrogenase at the transcript level was the group B [FeFe]-hydrogenase (**Supplementary Table 6**). It was followed by B9W14_RS14610- RS14620 encoding a trimeric group A3 [FeFe]-hydrogenase and by B9W14_RS11005-RS11015 encoding the group 1a [NiFe] hydrogenase, and, at a lower transcript level, B9W14_RS20180-RS20205 corresponding to the hexameric group A4 [FeFe]-hydrogenase.

[FeFe]-hydrogenase maturation proteins

The assembly of active [FeFe]-hydrogenases follows a complex pathway, where the [4Fe–4S]$_H$ cluster is installed by the regular Fe–S cluster assembly whereas the construction of the [2Fe]$_H$ cluster and its linkage to the [4Fe–4S]$_H$ involves three proteins HydE, HydF and HydG (47, 48). The engagement of this set of proteins in [FeFe]-hydrogenases' maturation was initially identified in *C. reinhardtii* (11). Disrupting *hydEF* resulted in a mutant incapable of assembling an active [FeFe]-hydrogenase and abolished H_2 production. Hydrogen production was restored after complementation of the mutant with wild-type genomic DNA containing the *hydEF* gene. Evidence supporting the conclusion that the HydEF and HydG proteins are required for the formation of an active [FeFe]-hydrogenase is shown by the heterologous expression of an active *C. reinhardtii* HydA1 protein in *E. coli*, a bacterium that lacks a native [Fe]-hydrogenase. The expression of the *hydA1* construct alone or co-expression of the *hydA1* and *hydEF* or *hydA1* and *hydG* genes in *E. coli* all resulted in the expression of non-functional HydA1 protein after purification. However, the co-expression of *C. reinhardtii hydA1* along with both *hydEF* and *hydG* in anaerobic *E. coli* cultures yielded an active HydA1 enzyme (11). *In vitro* activation of a [FeFe]-hydrogenase achieved combining *E. coli* cell extracts containing the heterologously expressed inactive *C*. *saccharobutylicum* HydA with extracts in which the maturation proteins HydE, HydF, and HydG were expressed individually or in varying combinations confirmed that the formation of active [FeFe]-hydrogenases requires the concerted action of HydE, HydF and HydG (8).

[NiFe]-hydrogenase maturation proteins

Like for the [FeFe] hydrogenases, for the [NiFe]-hydrogenase maturation a large number of studies have been made over the years to elucidate the mechanism and the role of each maturation protein.

The metallochaperone HypA brings about the HypB-dependent Ni acquisition cycle in [NiFe]-hydrogenase maturation (19, 49, 50). The HypA monomeric structure is composed of a Nickel and a Zinc metal binding domain (19), both featuring conserved residues (17, 51).

HypB is also a metallochaperone, with a characteristic GTP (52, 20) or ATPase (53) activity essential for the maturation. Therefore, HypB harbors the conserved GTP or ATP domain typical of these classes, with the related conserved residues (19, 20).

The small metallochaperone HypC is involved in the Iron insertion in the premature large subunit. These proteins have a strongly conserved cysteine in the second position (N-terminal), which is important for the interactions with the large subunit and its partner HypD, and a conserved histidine (19), which recurs in position 45 in our *Clostridium* acetogens.

The Iron-Sulfur protein HypD acts as a scaffold for the formation of the Fe(CN)2CO moiety. The central portion of its structure is characterized by four conserved motifs containing cysteines essential for the maturation (54), while its C-terminal is characterized by an atypical conserved motif responsible to coordinate the [4Fe-4S] cluster. We found that in one of the four conserved motifs mentioned above, with the exception of one putative homologous of *C. carboxidivorans* Ccar_RS06370, the latter serine (Ser) in P-x-H-V-S is always substitutes by a His.

HypE and HypF are together involved in the synthesis of the CN^- ligand. HypE is a carbamoyl dehydratase, an enzyme member of the PurM (aminoimidazole ribonucleotide synthetase) family, which catalyzes an ATP-dependent conversion of acyl compounds. Essential for its function is the conserved C-terminal tail that contains a conserved cysteine (19), which is also the substrate for his partner HypF. The carbamoyltransferase

HypF, the largest Hyp protein, is composed of four distinct domains, one of which is a typical zinc finger with a conserved binding motif.

Supplementary Tables and Figures

Supplementary File 1. Survey of predicted hydrogenase catalytic subunits in acetogens of the genus *Clostridium*. Sheet 1 contains the list of putative catalytic subunits resulted from the alignment-based sequence analysis. Columns A-F: information about the candidate hydrogenase catalytic subunit; columns H-X: structural domain analysis and functional annotation of the subunits predicted for each identified hydrogenase; columns Z-AC: fulfillment of the criteria adopted to validate the catalytic subunit (large and small subunits for putative [NiFe]-hydrogenases). Sheet 2 displays the amino acid positions of the metal binding motifs supporting the validation of the catalytic subunits of the [FeFe]-hydrogenases (columns S-U and BN-CE) and the large subunits of the [NiFe]-hydrogenases (columns C-R and V-BM). Sheet 3 displays the amino acid positions of the metal binding motifs supporting the presence of at least one Fe-S cluster in the [NiFe]-hydrogenases' small subunits which were identified by structural domain annotation.

Supplementary Table 1. Summary of percent sequence identity among hydrogenase catalytic subunits classified by HydDB within each subgroup.

HYD class	Minimum	1st Quartile	Median	Mean	3rd Quartile	Maximum
[NiFe] 1a	20,16	34,63	38,38	41,57	43,74	99,81
[NiFe] 1b	37,08	44,52	47,97	52,24	58,63	99,82
[NiFe] 1c	46,03	55,38	58,88	63,36	72,57	99,65
[NiFe] 1d	19,3	51,22	57,82	59,61	66,99	99,83
[NiFe] 1e	48,72	58,91	68,84	66,73	72,14	99,3
[NiFe] 1f	37,04	47,66	52,16	55,87	63,44	99,28
[NiFe] 1g	43,29	49,29	51,54	61,47	81,06	98,9

[NiFe] 1h	58,35	71,67	75	76,22	79,48	99,83
[NiFe] 1i	49,03	63,7	67,02	67,81	71,28	99,82
[NiFe] 1j	58,97	61,89	64,55	66,36	70,07	80,31
[NiFe] 1k	34,28	36,47	50,72	51.3	59,13	97,12
[NiFe] 1l	19,4	47,61	51,13	52,52	55,59	86,57
[NiFe] 2a	37,5	55,24	57,17	64,15	74,48	99,62
[NiFe] 2b	26,09	52,6	55,16	56,19	57,97	99,79
[NiFe] 2c	37,9	42,21	46,37	50,6	57,65	99,8
[NiFe] 2d	31,28	35,46	38,6	41,12	43,12	100
[NiFe] 2e	55,67	56,06	57,26	62,33	69,87	74,07
[NiFe] 3a	24,69	36,01	39,7	44,54	46,68	96,83
[NiFe] 3b	22,54	33,57	36,74	41,39	48	99,77
[NiFe] 3c	22,41	39,48	42,39	44,94	46,84	99,6
[NiFe] 3d	24,72	44,8	48,43	53,28	60,25	99,8
[NiFe] 4a	27,59	61,63	70,61	68,53	74,4	99,83
[NiFe] 4b	24	46,06	48,95	51,56	54,21	99,82
[NiFe] 4c	40	53,6	57,14	58,7	62,88	99,72
[NiFe] 4d	24,05	45,76	47,57	55,45	70,43	95,76
[NiFe] 4e	28,57	51,65	56,35	56,04	61,22	100
[NiFe] 4f	26,19	45,42	47,81	49,41	51,04	99,81
[NiFe] 4g	23,53	42,28	44,57	46,08	46,52	94,8
[NiFe] 4h	22,45	48,31	51,35	56,58	67,68	96,22
[NiFe] 4i	45,11	50	54,08	62,15	73,12	95,99
[FeFe] A1	16,29	40,79	44,8	45,35	48,77	100
[FeFe] A2	22,81	46,56	52,73	53,64	59,48	100
[FeFe] A3	21,15	46,46	51,27	51,61	56,25	100
[FeFe] A4	33,62	53,18	60,45	59,78	65	100
[FeFe] B	23,03	36,21	38,98	41,24	42,77	100
[FeFe] C1	25,7	38,04	44,32	45,67	50	100
[FeFe] C2	22,39	36,36	40	41,96	46,52	77,71
[FeFe] C3	20,11	31,76	34,88	35,98	38,73	100

Supplementary Table 2. Table displaying the subcellular localization for the small subunits of the group 1a [NiFe]-hydrogenases predicted in the clostridial acetogens considered in this study. The table reports the results of each of PSORTb's analytical modules and the confidence values for each of the localization sites. If one of the sites has a score of 7.5 or greater, the inputted protein is assigned to this site.

[NiFe]-hydrogenase small subunit	Cytoplasmic	Cytoplasmic membrane	Cell wall	Extracellular	CMSVM+	CWSVM+	CytoSVM+	ECSVM+U	ModHMM+	Motif+	Profile+	SCL-BLAST+	SCL-BLASTe+	Signal+
CLAU_RS04065	1.05	8.78	0.08	0.09	unknown	unknown	cytoplasmic	unknown	unknown	unknown	unknown	cytoplasmic membrane	unknown	unknown
Ccar_RS06385	0.17	9.51	0.16	0.15	unknown	unknown	unknown	unknown	unknown	unknown	unknown	cytoplasmic	unknown	unknown

												membrane		
CLJU_RS14145	1.05	8.78	0.08	0.09	unknown	unknown	cytoplasmic	unknown	unknown	unknown	unknown	cytoplasmic membrane	unknown	unknown
DMR38_RS14550	1.05	8.78	0.08	0.09	unknown	unknown	cytoplasmic	unknown	unknown	unknown	unknown	cytoplasmic membrane	unknown	unknown
B9W14_RS11010	1.05	8.78	0.08	0.09	unknown	unknown	cytoplasmic	unknown	unknown	unknown	unknown	cytoplasmic membrane precursor])	unknown	unknown
Csca_RS07905	1.05	8.78	0.08	0.09	unknown	unknown	cytoplasmic	unknown	unknown	unknown	unknown	cytoplasmic membrane	unknown	unknown
CLRAG_RS17290	1.05	8.78	0.08	0.09	unknown	unknown	cytoplasmic	unknown	unknown	unknown	unknown	cytoplasmic membrane	unknown	unknown
CLCOS_RS05465	1.05	8.78	0.08	0.09	unknown	unknown	cytoplasmic	unknown	unknown	unknown	unknown	cytoplasmic membrane	unknown	unknown

Supplementary Table 3. Sequences producing significant alignments against the System I and System II genes in charge of cytochrome c biosynthesis. The query sequences for *ccsA* and *ccsB* of the System II pathway are obtained from *Chlamydomonas Reinhardtii P48269* and *Bordetella pertussis*, respectively, while the query sequences for the *ccm* genes of the System I pathway are obtianed from either *Rhodobacter capsulatus* (*R. capsulatus*) or *Escherichia coli* (*E. coli*). The table reports the UniprotKB identifiers of the query genes. The query genes extracted from *R. capsulatus* and *E. coli* along the corresponding hits are colored in blue and orange, respectively.The table displays whether the blastp alignment returned acetogenic sequences featuring significant similarity (E-value < 0.05). The table displays, for each hit, the obtained query coverage, E-value and percent identity values.

**Cytochrome c**	***C. autoethanogenum***	***C. carboxidivorans***	***C. coskatii***	***C. drakei***	***C. ljungdahlii***	***C. ragsdalei***	***C. scatologenes***	***C.* sp. AWRP**

**biosynthesis genes**									
**System II (ccs genes)**									
***ccsA* |P48269 (*Chlamydomonas reinhardtii*)**		No hit	No hit	No hit	No hit	No hit	No hit	No hit	No hit
***ccsB* | A0A380ZWG6 (*Bordetella pertussis*)**		Best hit: CLAU_RS12925 Query cover: 11% E-value: 0,019 Per ident: 22,78%	Ccar_RS17780 Query cover: 6% E-value: 0,004 Per ident: 35,42%	CLCOS_RS00385 Query cover: 11% E-value: 0,014 Per ident: 22,78%	No hit	CLJU_RS02750 Query cover: 11% E-value: 0,015 Per ident: 22,78%	CLRAG_RS03295 Query cover: 11% E-value: 0,032 Per ident: 22,78%	No hit	DMR38_RS02475 Query cover: 11% E-value: 0,047 Per ident: 22,78%
**System I (ccm genes)**									
***ccmA* | P29959 (*Rhodobacter capsulats*)**	***ccmA* | P33931 (*Escherichia coli*)**	Best hit: CLAU_RS03305 |CLAU_RS03700 Query cover: 81% | 20% E-value: 3e-18 | 2e-20 Per ident: 27,32% | 28,72%	Best hit: Ccar_RS08525 | Ccar_RS13590 Query cover: 85% |21% E-value: 4e-21 |6e-08 Per ident: 29,50% |27,18%	Best hit: CLCOS_RS08450 |CLCOS_RS01255 Query cover: 81% |20% E-value: 2e-18 |9e-22 Per ident: 27,84% |27,89%	Best hit: B9W14_RS13080 |B9W14_RS06675 Query cover: 85% |20% E-value: 4e-21 |1e-18 Per ident: 30,50% |28,27%	Best hit: CLJU_RS12945 |CLJU_RS13340 Query cover: 81% |20% E-value: 4e-18 |2e-20 Per ident: 27,32% |28,72%	Best hit: CLRAG_RS00415 |CLRAG_RS04325 Query cover: 71% |95% E-value: 3e-18 |4e-23 Per ident: 31,93% |27,50%	Best hit: No hit | Csca_RS00850 Query cover: n.a. | 22% E-value: n.a. | 3e-20 Per ident: n.a. |28%	Best hit: DMR38_RS09120 | DMR38_RS10045 Query cover: 81% |21% E-value: 8e-10 |6e-21 Per ident: 27,84% |28,14%
***ccmB* | P29960 (*Rhodobacter capsulats*)**	***ccmB* | P0ABL8 (*Escherichia coli*)**	Best hit: No hit |CLAU_RS03700 Query cover: n.a. |20% E-value: n.a. |2e-20 Per ident: n.a. |28,72%	Best hit: No hit | Ccar_RS13590 Query cover: n.a. | 21% E-value: n.a. | 6e-18 Per ident: n.a. | 27,18%	Best hit: No hit | CLCOS_RS01255 Query cover: n.a. |20% E-value: n.a. | 9e-22 Per ident: n.a. | 27,89%	Best hit: No hit |B9W14_RS06675 Query cover: n.a. | 20% E-value: n.a. | 1e-18 Per ident: n.a. | 28,27%	Best hit: No hit | CLJU_RS13340 Query cover: n.a. | 20% E-value: n.a. | 2e-20 Per ident: n.a. | 28,72%	Best hit: No hit |CLRAG_RS04325 Query cover: n.a. | 20% E-value: n.a. | 5e-20 Per ident: n.a. | 28,72%	Best hit: Csca_RS22690 | Csca_RS00850 Query cover: 87% | 22% E-value: 2e-20 | 3e-20 Per ident: 26,83% |28,00%	Best hit: No hit | DMR38_RS21725 Query cover: n.a. | 7% E-value: n.a. | 0,047 Per ident: n.a. | 28,89%
***ccmC* | P29961 (*Rhodobacter capsulats*)**	***ccmC* | P0ABM1 (*Escherichia coli*)**	No hit	No hit	No hit	No hit	No hit	No hit	No hit	No hit
ccmD* | P29963 (*Rhod	***ccmD* | P0ABM5 (*Esch***	No hit	No hit	No hit	No hit	No hit	No hit	No hit	No hit

<table>
<tr><td>obacter capsulats)</td><td>erichia coli)</td><td></td><td></td><td></td><td></td><td></td><td></td><td></td><td></td></tr>
<tr><td>ccmE | Q9LA01 (Rhodobacter capsulats)</td><td>ccmE | P69490 (Escherichia coli)</td><td>No hit</td><td>No hit</td><td>Best hit: No hit | CLCOS_RS19355
Query cover: n.a. | 51%
E-value: n.a. | 0,031
Per ident: n.a. | 23,08%</td><td>No hit</td><td>Best hit: No hit | CLJU_RS11295
Query cover: n.a. | 51%
E-value: n.a. | 0.015
Per ident: n.a. | 23,08%</td><td>No hit</td><td>No hit</td><td>No hit</td></tr>
<tr><td>ccmF | Q00500 (Rhodobacter capsulats)</td><td>ccmF | P33927 (Escherichia coli)</td><td>No hit</td><td>No hit</td><td>No hit</td><td>No hit</td><td>No hit</td><td>No hit</td><td>No hit</td><td>No hit</td></tr>
<tr><td>ccmG | P36893 (Rhodobacter capsulats)</td><td>ccmG | P0AA86 (Escherichia coli)</td><td>Best hit: CLAU_RS09120 | n.a.
Query cover: 9% | n.a.
E-value: 0,003 | n.a.
Per ident: 58,82% | n.a.</td><td>Best hit: Ccar_RS11985 | Ccar_RS11985
Query cover: 81% | 57%
E-value: 3e-08 | 1e-08
Per ident: 22,98% | 24,79%</td><td>Best hit: CLCOS_RS10705 | n.a.
Query cover: 9% | n.a.
E-value: 0,003 | n.a.
Per ident: 58,82% | n.a.</td><td>Best hit: B9W14_RS16190 | B9W14_RS16190
Query cover: 60% | 57%
E-value: 6e-10 | 5e-08
Per ident: 25,64% | 24,79%</td><td>Best hit: CLJU_RS19945 | n.a.
Query cover: 9% | n.a.
E-value: 0,003 | n.a.
Per ident: 58,82% | n.a.</td><td>Best hit: CLRAG_RS11180 | n.a.
Query cover: 55% | n.a.
E-value: 8e-04 | n.a.
Per ident: 22,68% | n.a.</td><td>Best hit: Csca_RS02335 | Csca_RS02335
Query cover: 63% | 57%
E-value: 5e-08 | 1e-07
Per ident: 23,77% | 23,93%</td><td>Best hit: DMR38_RS20050 | n.a.
Query cover: 55% | n.a.
E-value: 0,001 | n.a.
Per ident: 22,68% | n.a.</td></tr>
<tr><td>ccmH | Q00501 (Rhodobacter capsulats)</td><td>ccmH | P0ABM9 (Escherichia coli)</td><td>No hit</td><td>No hit</td><td>No hit</td><td>No hit</td><td>No hit</td><td>No hit</td><td>No hit</td><td>No hit</td></tr>
<tr><td>ccmI | A0A4U1JTZ4 (Rhodobacter capsulats)</td><td>//</td><td>Best hit: n.a. | Ccar_RS02950
Query cover: n.a. | 10%
E-value: n.a. | 0,01
Per ident: n.a. | 44,44%</td><td>No hit</td><td>No hit</td><td>No hit</td><td>No hit</td><td>No hit</td><td>No hit</td><td>Best hit: No hit | DMR38_RS20920
Query cover: n.a. | 12%
E-value: n.a. | 0,035
Per ident: n.a. | 37,78%</td></tr>
<tr><td>ccdA |</td><td>//</td><td>No hit</td><td>No hit</td><td>No hit</td><td>No hit</td><td>No hit</td><td>No hit</td><td>No hit</td><td>No hit</td></tr>
</table>

**D5AVB1 (*Rhodobacter capsulats*)**								
***dsbD* | P36655 (*Escherichia coli*)**	Best hit: CLAU_RS09005 Query cover: 32% E-value: 2e-05 Per ident: 26,56%	Best hit: Ccar_RS11985 Query cover: 54% E-value: 5e-08 Per ident: 20,94%	Best hit: CLCOS_RS00940 Query cover: 32% E-value: 4e-05 Per ident: 26,32%	Best hit: B9W14_RS16190 Query cover: 30% E-value: 1e-05 Per ident: 21,55%	Best hit: CLJU_RS19815 Query cover: 32% E-value: 2e-05 Per ident: 26,56%	Best hit: CLRAG_RS20460 Query cover: 7% E-value: 0,046 Per ident: 28,89%	Best hit: Csca_RS02335 Query cover: 31% E-value: 3e-06 Per ident: 21,93%	Best hit: DMR38_RS21725 Query cover: 7% E-value: 0,047 Per ident: 28,89%

Supplementary Table 4. Sequences producing significant alignments against the coproporphyrin-dependent pathway in charge of cytochrome b biosynthesis. The query sequences for the genes belonging to the coproporphyrin-dependent pathway in charge of cytochrome b biosynthesis are obtained from *Escherichia coli*. The table reports the UniprotKB identifiers of the query genes. The table displays whether the blastp alignment returned acetogenic sequences featuring significant similarity (E-value < 0.05). The table displays, for each hit, the obtained query coverage, E-value and percent identity values.

**Cytochrome cb biosynthesis genes**	***C. autoethanogenum***	***C. carboxidivorans***	***C. coskatii***	***C. drakei***	***C. ljungdahlii***	***C. ragsdalei***	***C. scatologenes***	***C.* sp. AWRP**
***cgoX* | P32397 (*Escherichia coli*)**	Best hit: CLAU_RS02210 Query cover: 10% E-value: 0.013 Per ident: 31.91%	Best hit: Ccar_RS10170 Query cover: 11% E-value: 0,028 Per ident: 39,62%	Best hit: CLCOS_RS14245 Query cover: 11% E-value: 0,005 Per ident: 35,19%	Best hit: B9W14_RS03765 Query cover: 10% E-value: 0,020 Per ident: 35,42%	Best hit: CLJU_RS11865 Query cover: 11% E-value: 0,005 Per ident: 35,19%	Best hit: CLRAG_RS08420 Query cover: 10% E-value: 0,020 Per ident: 37,50%	Best hit: Csca_RS15530 Query cover: 10% E-value: 0,021 Per ident: 35,42%	Best hit: DMR38_RS12325 Query cover: 11% E-value: 0,004 Per ident: 37,04%
***uroD* | P32395 (*Escherichia coli*)**	Best hit: CLAU_RS19765 Query cover: 53% E-value: 5e-10 Per ident:	Best hit: Ccar_RS24745 Query cover: 81% E-value: 1e-09 Per ident:	Best hit: CLCOS_RS20505 Query cover: 53% E-value: 5e-10 Per ident:	Best hit: B9W14_RS03735 Query cover: 60% E-value: 3e-11 Per ident:	Best hit: CLJU_RS09375 Query cover: 53% E-value: 6e-09 Per ident:	Best hit: CLRAG_RS15200 Query cover: 61% E-value:2e-08 Per ident:	Best hit: Csca_RS15585 Query cover: 60% E-value: 3e-11 Per ident:	Best hit: DMR38_RS10780 Query cover: 61% E-value: 2e-08 Per ident:

	27,98%	25,49%	27,98%	29,03%	26,63%	26,61%	29,03%	26,07
***cpfc* | P32396 (*Escherichia coli*)**	No hit	No hit	No hit	No hit	Best hit: CLJU_RS15 345 Query cov: 61% E-value: 0.031 Per ident: 24,55%	No hit	No hit	No hit
***chdc* | Q2G0J1 (*Escherichia coli*)**	No hit	No hit	No hit	No hit	No hit	No hit	No hit	No hit
***cdhH* | P39645 (*Escherichia coli*)**	No hit	Best hit: Ccar_RS26 400 Query cover: 33% E-value: 0,015 Per ident: 25,58%	No hit	No hit	No hit	No hit	No hit	No hit

Supplementary Table 5. Survey of transcript-level expression values for the hydrogenases predicted in *C. ljungdahlii*. The table displays the FPKM values of individual hydrogenase subunits, as reported in the RNA-seq analysis carried out in cell cultures grown on H_2/CO_2 [10].

**Hydogenase classification**	**Locus tag (RefSeq)**	**Locus tag (GenBank)**	**Nagarajan et al. 2013** H_2/CO_2 FPKM
**NiFe 1a**	CLJU_RS14140	CLJU_c28660	0,345
	CLJU_RS14145	CLJU_c28670	0
**FeFe B**	CLJU_RS09955	CLJU_c20290	10,947
**FeFe A4**	CLJU_RS03460	CLJU_c07030	732,5
	CLJU_RS03465	CLJU_c07040	619,663
	CLJU_RS03470	CLJU_c07050	1463,94
	CLJU_RS03475	CLJU_c07060	2066,86
	CLJU_RS03480	CLJU_c07070	2808,01
	CLJU_RS03485	CLJU_c07080	4501,23
**FeFe A3**	CLJU_RS07205	CLJU_c14700	4,18
	CLJU_RS07210	CLJU_c14710	1,627
	CLJU_RS07215	CLJU_c14720	0
**FeFe A4**	CLJU_RS08480	CLJU_c17270	9,17
	CLJU_RS08485	CLJU_c17280	12,906
	CLJU_RS08490	CLJU_c17290	13,793

Supplementary Table 6. Survey of transcript-level expression values for the hydrogenases predicted in *C. drakei*. The table displays the FPKM values of individual hydrogenase subunits, as reported in the RNA-seq analysis carried out in cell cultures grown on H_2/CO_2 [12].

Hydrogenase classification	Locus tag (RefSeq)	Locus tag (GenBank)	Song et al. (2020) 80% H_2, 20% CO_2
FeFe_A3	B9W14_RS06375	B9W14_06375	0
	B9W14_RS06380	B9W14_06380	8,7
	B9W14_RS06385	B9W14_06385	2,31
NiFe_1a	B9W14_RS11015	B9W14_11015	19,98
	B9W14_RS11010	B9W14_11010	55,71
	B9W14_RS11005	B9W14_11005	147,64
FeFe_A3	B9W14_RS14620	B9W14_14620	120,08
	B9W14_RS14615	B9W14_14615	89,61
	B9W14_RS14610	B9W14_14610	53,94
FeFe_A4	B9W14_RS20185	B9W14_20185	0
	B9W14_RS20180	B9W14_20180	0
	B9W14_RS20190	B9W14_20190	18,6
	B9W14_RS20195	B9W14_20195	48,19
	B9W14_RS20200	B9W14_20200	95,48
	B9W14_RS20205	B9W14_20205	29,61
FeFe_A3	B9W14_RS20215	B9W14_20215	97,06
	B9W14_RS20220	B9W14_20220	0,53
	B9W14_RS20225	B9W14_20225	2,5
FeFe B	B9W14_RS24170	B9W14_24170	160,5

Supplementary Table 7. Amino acid positions of the motifs supporting the identification of the genes encoding the rSAM [FeFe]-hydrogenases maturation proteins, HydG and HydE.

Gene	Microorganism	RefSeq ID	*N-terminal Radical-SAM signature* (Cx_3Cx_2C)	*C-terminal FeS cluster signature* (Cx_7Cx_2C)	Conserved YxxY
hydE	*C. autoethanogenum*	CLAU_RS08145	64-71	312-323	304-307
	C. carboxidivorans	Ccar_RS19085	63-70	311-322	303-306
	C. ljungdahlii	CLJU_RS18880	64-71	312-323	304-307
	C. sp. AWRP	DMR38_RS19105	64-71	312-323	304-307
	C. coskati	CLCOS_RS19995	64-71	312-323	304-307
	C. ragsdalei	CLRAG_RS10265	64-71	312-323	304-307

	C. drakei	B9W14_RS22750	63-70	311-322	303-306
	C. scatologenes	Csca_RS21720	63-70	311-322	303-306

Gene	Microorganism	RefSeq ID	***N-terminal Radical-SAM signature*** **(Cx_3Cx_2C)**	***C-terminal FeS cluster signature*** **($Cx_2Cx_{22}C$)**	**C-terminal FeS cluster signature ($Cx_5Cx_{19}C$)**
hydG	*C. Scatologenes*	Csca_RS08575	96-103	386-412	/
	C. drakei	B9W14_RS10345	96-103	386-412	/
	C. carboxidivorans	/	/	/	/
	C. ljungdahlii	/	/	/	/
	C. autoethanogenum	CLAU_RS03260	109-116	/	386-412
	C. coskati	/	/	/	/
	C. ragsdalei	/	/	/	/
	C. sp. AWRP	/	/	/	/

Supplementary Table 8. Putative HydG proteins with atypical sequence features.

Microorganism	Putative *hydG*
C. carboxidivorans	Ccar_RS24800
C. ljungdahlii	CLJU_RS11845
C. sp. AWRP	DMR38_RS12345
C. coskati	CLCOS_RS17725
C. ragsdalei	CLRAG_RS15175
C. scatologenes	Csca_RS15510
C. drakei	B9W14_RS03785

Supplementary Table 9. Amino acid positions of the motifs supporting the identification of genes encoding HydF.

Gene	Microorganism	RefSeq ID	***P-loop N-terminal (GxxxxGKS/T)***	**Walker B Mg2+ binding (hhhhDxxG)**	**Distal, GTP binding motif (N/TKxD)**	***C-terminal FeS cluster signature ($CxHx_{46-53}CxxC$)***
hydF	*C. autoethanogenum*	CLAU_RS10040	18-25	62-69	127-130	302-356
	C. carboxidivorans	Ccar_RS05080	19-26	63-70	128-131	307-359
	C. ljungdahlii	CLJU_RS20880	18-25	62-69	127-130	306-358
	C. coskati	CLCOS_RS18855	18-25	62-69	127-130	306-358
	C. drakei	B9W14_RS10005	19-26	63-70	128-131	307-359
	C. ragsdalei	CLRAG_RS02690	18-25	62-69	127-130	306-358
	C. scatologenes	Csca_RS08935	19-26	63-70	128-131	307-359

	C. sp. AWRP	DMR38_RS21245	18-25	62-69	127-130	306-358

Supplementary Figure 1. The evolutionary tree was inferred using the Maximum Likelihood method and JTT matrix-based model [27] of the MEGA-X software [28] with the amino acid sequences of the hydrogenases validated in the acetogens of genus *Clostridium*. Figure shows the tree with the highest log likelihood. The percentage of trees in which the associated genes clustered together is shown next to the branches. Initial tree(s) for the heuristic search were obtained automatically by applying Neighbor-Join and BioNJ algorithms to a matrix of pairwise distances estimated using the JTT model, and then selecting the topology with superior log likelihood value. The tree is drawn to scale, with branch lengths measured in the number of substitutions per site.

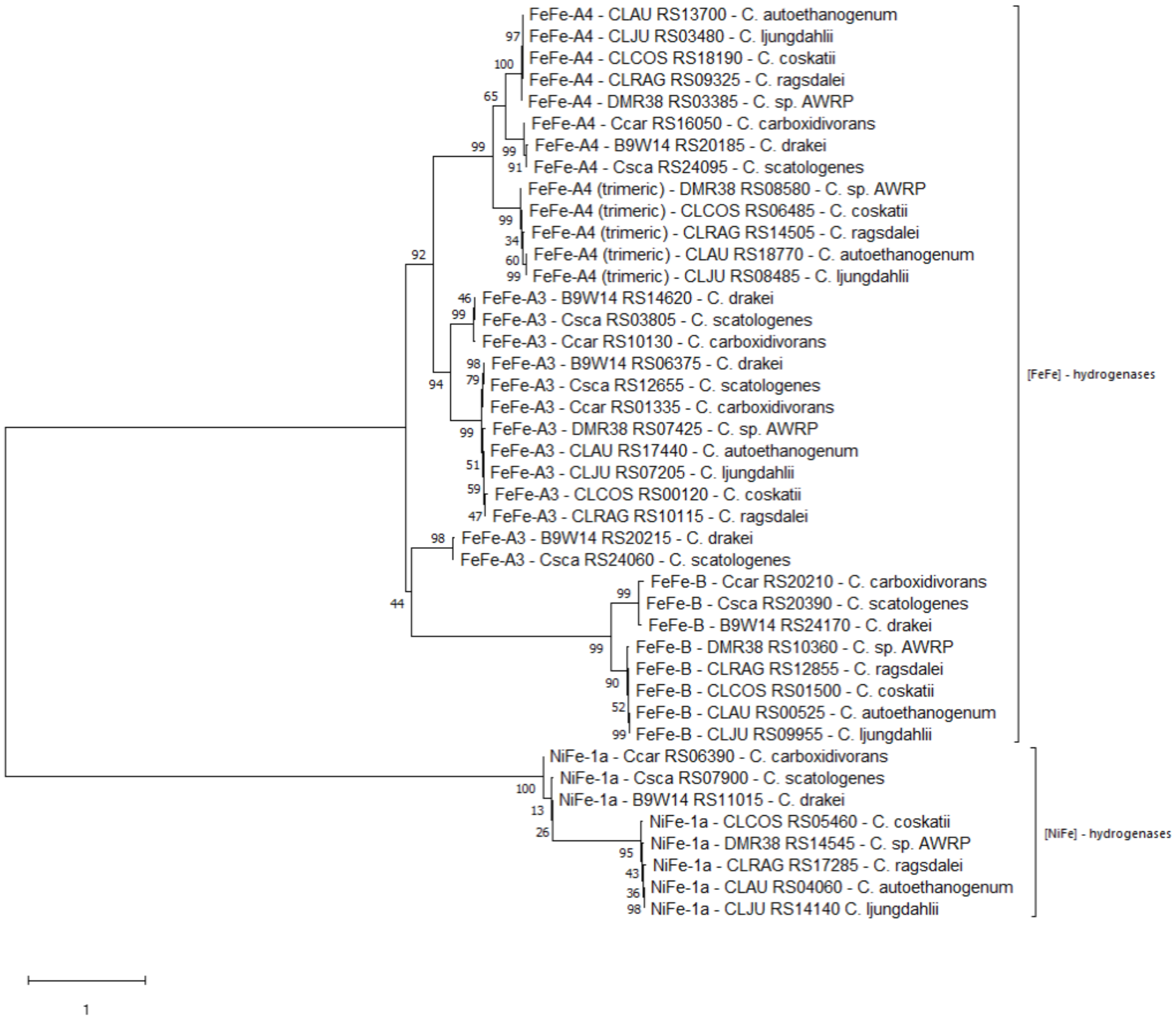

Supplementary Figure 2. Multiple sequence alignment of the atypical HydG proteins listed in Supplementary Table 9. Light blue: conserved cysteine of a putative N-terminal rSAM signature Cx_3Cx_2C; orange: conserved cysteine of a putative C-terminal Fe-S cluster.

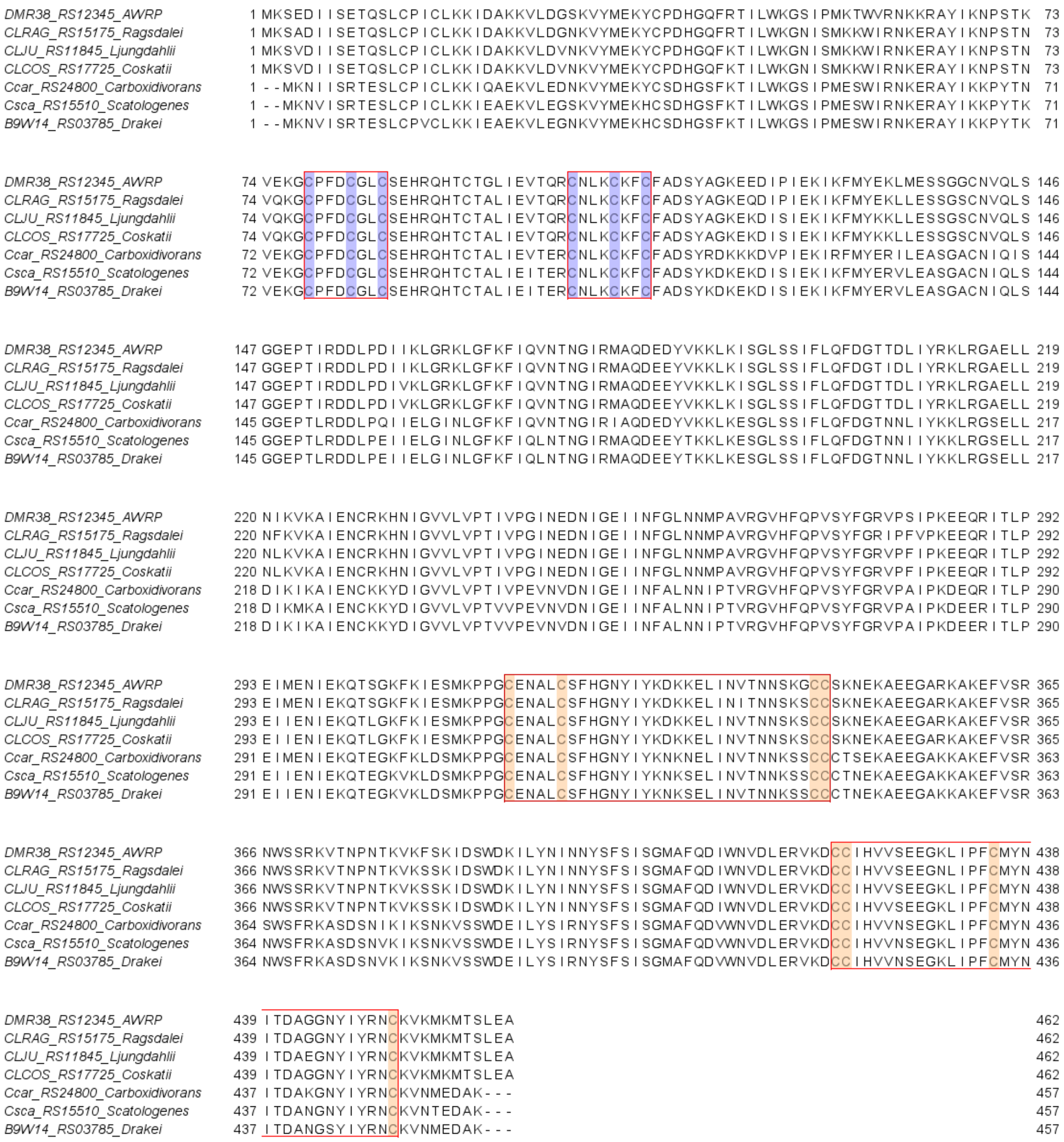

Supplementary Figure 3. Full alignment of NuoF from *T. thermophilus* with the cofactor binding subunits of the hexameric [FeFe]-hydrogenases of group A4. Red boxes: amino acids of the two conserved loops involved in the interaction with FMN and NAD/NADP; blue: α helices; yellow: β sheets.

```
NuoF_Thermus_thermophilus          1 ------------------------------------------------------MTGPIL-----------   6
A4_03370_AWRP                      1 MSDKKTVNICCGTGCLAKGSMEVYEEMKAQVAKLGANAEV--NVTLKATGCDGLCEKGPVLKIYPDDIAYFKV  71
A4_13685_Autoethanogenum           1 MSDKKTVNICCGTGCLAKGSMEVYEEMKAQIAKLGANAEV--NVKLKATGCDGLCEKGPVLKIYPDDIAYFKV  71
A4_03465_Ljungdahlii               1 MSDKKTVNICCGTGCLAKGSMEVYEEMKAQIAKLGANAEV--NVKLKATGCDGLCEKGPVLKIYPDDIAYFKV  71
A4_18175_Coskatii                  1 MSDKKTVNICCGTGCLAKGSMEVYEEMKAQIAKLGANAEV--NVKLKATGCDGLCEKGPVLKIYPDDIAYFKV  71
A4_09310_Ragsdalei                 1 MSDKKIVNICCGTGCLAKGSKEVYEEMKAQIAKLGANAEV--NVKLKATGCDGLCEKGPVLKIYPDDIAYFKV  71
A4_16065_Carboxidivorans           1 MSNKKIVNVCCGAGCLAKHSMEVFEELKKKVAELGANAEVKTEVQLKATGCDGLCEKGPVIKIYPDDIAYFKV  73
A4_20200_Drakei                    1 MSNKKTVNVCCGAGCLAKHSMEVFEELKKKVAEMGANAEVKTEVKLKATGCDGLCEKGPVIKIYPDDIAYFKV  73
A4_24080_Scatologenes              1 MSNKKTVNVCCGAGCLAKHSMEVFEELKKKVAELGANAEVKTEVKLKATGCDGLCEKGPVIKIYPDDIAYFKV  73

NuoF_Thermus_thermophilus          7 --------------------------------------SGLDPRFERTLYAHVGKEGSWTLDYYLRHGGYETA  41
A4_03370_AWRP                     72 KVEDVEDVVKKTLMNGEIIEKLLYFETATKQRLRNHKESEFCKRQYKIALRNVGEIDPISLEDYVERGGYKAL 144
A4_13685_Autoethanogenum          72 KVEDVEDVVKKTLMNGEIIEKLLYFETATKQRLRNHKESEFCKRQYKIALRNVGEIDPISLEDYVERGGYKAL 144
A4_03465_Ljungdahlii              72 KVEDVEDVVKKTLMNGEIIEKLLYFETATKQRLRNHKESEFCKRQYKIALRNVGEIDPISLEDYVERGGYKAL 144
A4_18175_Coskatii                 72 KVEDVEDVVKKTLMNGEIIEKLLYFETATKQRLRNHKESEFCKRQYKIALRNVGEIDPISLEDYVERGGYKAL 144
A4_09310_Ragsdalei                72 KVEDVEDVVKKTLMNGEIIEKLLYFETATKQRLRNHKESEFCKRQYKIALRNVGEIDPISLEDYVERGGYKAL 144
A4_16065_Carboxidivorans          74 KVEDVEDVVKKTLMNGEIIEKLLYFETSTKQRLRNHKQSEFCKRQNKIALRNVGEIDPTVIEDYIERGGYKAL 146
A4_20200_Drakei                   74 KVEDIEDVVKKTLMNGEIIEKLLYFETSTKQRLRNHKQSEFCKRQNKIALRNVGEIDPTVIEDYIERGGYKAL 146
A4_24080_Scatologenes             74 KVEDVEDVVKKTLMNGEIIEKLLYFETSTKQRLRNHKQSEFCKRQNKIALRNVGEIDPTVIEDYIERGGYKAL 146

NuoF_Thermus_thermophilus         42 KRVLKEKTPDEVIEEVKRSGLRGRGGAGFPTGLKWSFMPKDDGKQHYLICNADESEPGSFKDRYILEDVPHLL 114
A4_03370_AWRP                    145 KKAISSMKPEDVLEEITKSGLRGRGGAGFPTGRKWKTAADIDTSPIYVVCNGDEGDPGAFMDRSIMEGDPNSV 217
A4_13685_Autoethanogenum         145 KKAISSMKPEDVLEEITKSGLRGRGGAGFPTGRKWKTAADIDTSPIYVVCNGDEGDPGAFMDRSIMEGDPNSV 217
A4_03465_Ljungdahlii             145 KKAISSMKPEDVLEEITKSGLRGRGGAGFPTGRKWKTAADIDTSPIYVVCNGDEGDPGAFMDRSIMEGDPNSV 217
A4_18175_Coskatii                145 KKAISSMKPEDVLEEITKSGLRGRGGAGFPTGRKWKTAADIDTSPIYVVCNGDEGDPGAFMDRSIMEGDPNSV 217
A4_09310_Ragsdalei               145 KKAISSMKPEDVLEEITKSGLRGRGGAGFPTGRKWKTAADIDTSPIYVVCNGDEGDPGAFMDRSIMEGDPNSV 217
A4_16065_Carboxidivorans         147 KKVISQMKPEDVLKEVGDSGLRGRGGAGFPTSRKWQTAAEIDTTPKYVVCNGDEGDPGAFMDRSIMEGDPHSV 219
A4_20200_Drakei                  147 KKVLSKMKPEDVLKEVGDSGLRGRGGAGFPTARKWQTAAQINTTPKYVVCNGDEGDPGAFMDRSIMEGDPHSV 219
A4_24080_Scatologenes            147 KKVLSEMKPEDVLKEVGDSGLRGRGGAGFPTARKWQTAAQINTTPKYVVCNGDEGDPGAFMDRSIMEGDPHSV 219

NuoF_Thermus_thermophilus        115 IEGMILAGYAIRATVGYIYVRGEYRRAADRLEQAIKEARARGYLGKNLFGTDFSFDLHVHRGAGAYICGEETA 187
A4_03370_AWRP                    218 IEGMTLCAYAVGGTNGFAYIRDEYGLAVENMQKAINKAKEKNLLGNNILGTDFSFDIQIVRGGGAFVCGESTA 290
A4_13685_Autoethanogenum         218 IEGMTLCAYAVGGTNGFAYIRDEYGLAVENMQKAINKAKDENLLGNNILGTDFSFDIQIVRGGGAFVCGESTA 290
A4_03465_Ljungdahlii             218 IEGMTLCAYAVGGTNGFAYIRDEYGLAVENMQKAINKAKDENLLGNNILGTDFSFDIQIVRGGGAFVCGESTA 290
A4_18175_Coskatii                218 IEGMTLCAYAVGGTNGFAYIRDEYGLAVENMQKAINKAKDENLLGNNILGTDFSFDIQIVRGGGAFVCGESTA 290
A4_09310_Ragsdalei               218 IEGMTLCAYAVGGTNGFAYIRDEYGLAVENMQKAINKAKDENLLGNNILGTDFSFDIQIVRGGGAFVCGESTA 290
A4_16065_Carboxidivorans         220 IEGMTICAYAVGGTLGFAYIRDEYGLAVENMQKAINVARERGFLGENILGTNFSFDIQIVRGGGAFVCGESTA 292
A4_20200_Drakei                  220 IEGMTICAYAVGGSLGFAYIRDEYGLAVENMQKAIDVARERGLLGKNILNTDFSFDIQIVRGGGAFVCGESTA 292
A4_24080_Scatologenes            220 IEGMTICAYAVGGSLGFAYIRDEYGLAVENMQKAIDVARERGLLGKNILDTDFSFDIQIVRGGGAFVCGESTA 292

NuoF_Thermus_thermophilus        188 LMNSLEGLRANPRLKPPFPAQSGLWGKPTTINNVETLASVVPIMERGADWFAQMGT-EQSKGMKLYQISGPVK 259
A4_03370_AWRP                    291 LMSSIEGMVGEPRAKYIHTTEKGLWGQPTVLNNVETWANVPIIIEKGGDWYHSIGTMKDSKGTKVFSLVGKVK 363
A4_13685_Autoethanogenum         291 LMSSIEGMVGEPRAKYIHTTEKGLWGQPTVLNNVETWANVPIIIEKGGDWYHAIGTMEKSKGTKVFSLVGKVK 363
A4_03465_Ljungdahlii             291 LMSSIEGMVGEPRAKYIHTTEKGLWGQPTVLNNVETWANVPIIIEKGGDWYHAIGTMEKSKGTKVFSLVGKVK 363
A4_18175_Coskatii                291 LMSSIEGMVGEPRAKYIHTTEKGLWGQPTVLNNVETWANVPIIIEKGGDWYHAIGTMEKSKGTKVFSLVGKVK 363
A4_09310_Ragsdalei               291 LMSSIEGMVGEPRAKYIHTTEKGLWGQPTVLNNVETWANVPIIIEKGGDWYHAIGTMEKSKGTKVFSLVGKVK 363
A4_16065_Carboxidivorans         293 LMSSIEGMVGEPRAKYIHTTEKGLWGQPTVLNNVETWANIPIILDKGGQWYHSIGTMEKSKGTKVFSLVGKVK 365
A4_20200_Drakei                  293 LMSSIEGMVGEPRAKYIHTTEKGLWGQPTVLNNVETWANIPIILEKGGDWYHSIGSMEKSKGTKVFSLVGKVK 365
A4_24080_Scatologenes            293 LMSSIEGMVGEPRAKYIHTTEKGLWGQPTVLNNVETWANIPIILEKGGDWYHSIGSMEKSKGTKVFSLVGKVK 365

NuoF_Thermus_thermophilus        260 RPGVYELPMGTTFRELIYEWAGGPLE--PIQAIIPGGSSTPPLPFTEEVLDTPMSYEHLQAKGSMLGTGGVIL 330
A4_03370_AWRP                    364 NTGLVEVPMGTTLREIIYDIGGGVLNDRKFKAVQIGGPSGGCLP--SEYLDLPVDYDTLVKADSMMGSGGMIV 434
A4_13685_Autoethanogenum         364 NTGLVEVPMGTTLREIIYDIGGGVLNDRKFKAVQIGGPSGGCLP--AEYLDLPVDYDTLVKADSMMGSGGMIV 434
A4_03465_Ljungdahlii             364 NTGLVEVPMGTTLREIIYDIGGGVLNDRKFKAVQIGGPSGGCLP--AEYLDLPVDYDTLVKADSMMGSGGMIV 434
A4_18175_Coskatii                364 NTGLVEVPMGTTLREIIYDIGGGVLNDRKFKAVQIGGPSGGCLP--AEYLDLPVDYDTLVKADSMMGSGGMIV 434
A4_09310_Ragsdalei               364 NTGLVEVPMGTTLREIIYDIGGGVLNDRKFKAVQIGGPSGGCLP--SEYLDLPVDYDTLVKADSMMGSGGMIV 434
A4_16065_Carboxidivorans         366 NTGLVEVPMGTTLREIIFNIGGGVLNDRKFKAVQIGGPSGGCLP--EEYLDLPVDYDTLTKADSMMGSGGMIV 436
A4_20200_Drakei                  366 NTGLVEVPMGTTLREIIYDIGGGVLNDRKFKAVQIGGPSGGCLP--EEYLDLPVDYDTLTKADSMMGSGGMIV 436
A4_24080_Scatologenes            366 NTGLVEVPMGTTLREIIYDIGGGVLNDRKFKAVQIGGPSGGCLP--EEYLDLPVDYDTLTKADSMMGSGGMIV 436

NuoF_Thermus_thermophilus        331 IPERVSMVDAMWNLTRFYAHESCGKCTPCREGVAGFMVNLFAKIGTGQGEEKDVENLEALLPLIEGRSFCPLA 403
A4_03370_AWRP                    435 MDDRTCMVDVTRYYLSFLAEESCGKCVPCREGVKR-MLEILTDICNGDGKEGDIEELLEICSMTSKASLCSLG 506
A4_13685_Autoethanogenum         435 MDDRTCMVDVTRYYLSFLAEESCGKCVPCREGVKR-MLEILTDICNGDGKEGDIEELLEICSMTSKASLCSLG 506
A4_03465_Ljungdahlii             435 MDDRTCMVDVTRYYLSFLAEESCGKCVPCREGVKR-MLEILTDICNGDGKEGDIEELLEICSMTSKASLCSLG 506
A4_18175_Coskatii                435 MDDRTCMVDVTRYYLSFLAEESCGKCVPCREGVKR-MLEILTDICNGDGKEGDIEELLEICSMTSKASLCSLG 506
A4_09310_Ragsdalei               435 MDDRTCMVDVTRYYLSFLAEESCGKCVPCREGVKR-MLEILTDICNGDGKEGDIEELLEICSMTSKASLCSLG 506
A4_16065_Carboxidivorans         437 MDDRTCIVDVTRYYLGFLAEESCGKCVPCREGVKR-MLEIMTDICNGEGREGDLEELLEIASMSKEAALCSLG 508
A4_20200_Drakei                  437 MDDRTCIVDVTRYYLSFLAEESCGKCVPCREGVKR-MLEILTDICNGEGREGDIEELLEICSMAKDAALCSLG 508
A4_24080_Scatologenes            437 MDDRTCIVDVTRYYLSFLAEESCGKCVPCREGVKR-MLEILTDICNGEGREGDIEELLEICSMAKDAALCSLG 508

NuoF_Thermus_thermophilus        404 DAAVWPVKGSLRHFKDQYLALAREKR------------------------------------PVPRPSLWR-- 438
A4_03370_AWRP                    507 KSAPNPVKAAIRYFRDEFEEHIKNKRCRAGVCKKLTTFGIDADKCKGCDMCKKNCPADCITGEIKKPHSIDAD 579
A4_13685_Autoethanogenum         507 KSAPNPVIASIRYFRDEFEEHIKNKRCRAGVCKKLTTFGIDEDKCKGCDMCKKNCPADCITGEIKKPHTIDAD 579
A4_03465_Ljungdahlii             507 KSAPNPVIASIRYFRDEFEEHIKNKRCRAGVCKKLTTFGIDEDKCKGCDMCKKNCPADCITGEIKKPHTIDAD 579
A4_18175_Coskatii                507 KSAPNPVIASIRYFRDEFEEHIKNKRCRAGVCKKLTTFGIDEDKCKGCDMCKKNCPADCITGEIKKPHTIDAD 579
A4_09310_Ragsdalei               507 KSAPNPVKAAIRYFRDEFEEHIKNKRCRAGVCKKLTTFGIDQDKCKGCDMCKKNCPADCITGEIKKPHTIDAD 579
A4_16065_Carboxidivorans         509 KSAANPVLASIRYFRDEFEEHIRNKRCRAGVCKKLTTFVIDEDKCKGCDICKKNCAVDAITGDVKKPHVIDDE 581
A4_20200_Drakei                  509 KSAPNPVVASIRYFRDEFEEHIRNKRCRAGVCKKLTTFVIDTDKCKGCGMCSKDCAVDAITGDVKKPHVIDDD 581
A4_24080_Scatologenes            509 KSAPNPVVASIRYFRDEFEEHIRNKRCRAGVCKKLTTFVIDADKCKGCGMCSKNCAVDAITGDVKKPHVIDDD 581

NuoF_Thermus_thermophilus            --------------------
A4_03370_AWRP                    580 KCLRCGNCMNICKFDAVKVL                                                     599
A4_13685_Autoethanogenum         580 KCLRCGNCMNICKFDAVKVL                                                     599
A4_03465_Ljungdahlii             580 KCLRCGNCMNICKFDAVKVL                                                     599
A4_18175_Coskatii                580 KCLRCGNCMNICKFDAVKVL                                                     599
A4_09310_Ragsdalei               580 KCLRCGNCMNICKFDAVKVL                                                     599
A4_16065_Carboxidivorans         582 KCVRCGNCMNMCKFDAVKVK                                                     601
A4_20200_Drakei                  582 KCVRCGNCMNICKFDAVKVK                                                     601
A4_24080_Scatologenes            582 KCVRCGNCMNICKFDAVKVK                                                     601
```

Supplementary Figure 4. Full alignment of NuoF from *T. thermophilus* with the cofactor binding subunits of the trimeric [FeFe]-hydrogenases of group A3. Red boxes: amino acids of the two conserved loops involved in the interaction with FMN and NAD/NADP; blue: α helices; yellow: β sheets.

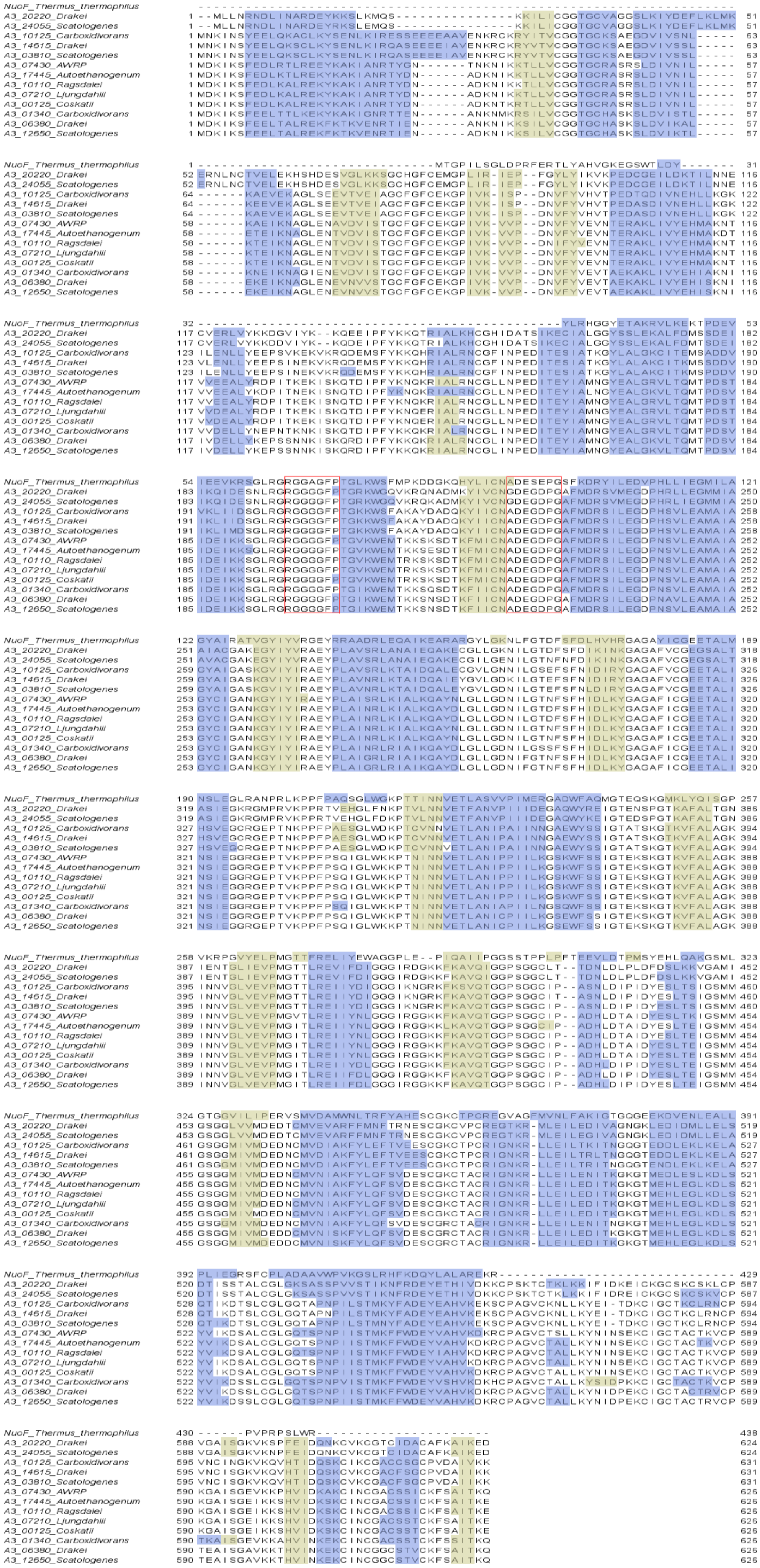
NuoF_Thermus_thermophilus
A3_20220_Drakei
A3_24055_Scatologenes
A3_10125_Carboxidivorans
A3_14615_Drakei
A3_03810_Scatologenes
A3_07430_AWRP
A3_17445_Autoethanogenum
A3_10110_Ragsdalei
A3_07210_Ljungdahlii
A3_00125_Coskatii
A3_01340_Carboxidivorans
A3_06380_Drakei
A3_12650_Scatologenes

References

References

1. **Kumar S, Stecher G, Li M, Knyaz C, Tamura K.** MEGA X: Molecular Evolutionary Genetics Analysis across Computing Platforms. Mol Biol Evol 2018; 35:1547-1549 doi: 10.1093/molbev/msy096
2. **Thompson JD, Higgins DG, Gibson TJ**. CLUSTAL W: improving the sensitivity of progressive multiple sequence alignment through sequence weighting, position-specific gap penalties and weight matrix choice. *Nucleic Acids Res* 1994; 22:4673-4680 doi: 10.1093/nar/22.22.4673
3. **Jones DT, Taylor WR, Thornton JM**. The rapid generation of mutation data matrices from protein sequences. *Comput Appl Biosci* 1992; 8:275-282 doi: 10.1093/bioinformatics/8.3.275
4. **King PW, Posewitz MC, Ghirardi ML, Seibert M**. Functional studies of [FeFe] hydrogenase maturation in an *Escherichia coli* biosynthetic system. *J Bacteriol* 2006; 188:2163-2172 doi: 10.1128/JB.188.6.2163-2172.2006
5. **Rubach JK, Brazzolotto X, Gaillard J, Fontecave M**. Biochemical characterization of the HydE and HydG iron-only hydrogenase maturation enzymes from Thermatoga maritima. FEBS Lett 2005; 579:5055-5060 doi: 10.1016/j.febslet.2005.07.092
6. **Duffus BR, Hamilton TL, Shepard EM, Boyd ES, Peters JW et al**. Radical AdoMet enzymes in complex metal cluster biosynthesis. Biochim Biophys Acta 2012; 1824:1254-1263 doi: 10.1016/j.bbapap.2012.01.002
7. **Shepard EM, Impano S, Duffus BR, Pagnier A, Duschene KS et al.** HydG, the "dangler" iron, and catalytic production of free CO and CN^-: implications for [FeFe]-hydrogenase maturation. Dalton Trans 2021; 50:10405-10422 doi: 10.1039/d1dt01359a
8. **McGlynn SE, Boyd ES, Shepard EM, Lange RK, Gerlach R et al.** Identification and characterization of a novel member of the radical AdoMet enzyme superfamily and implications for the biosynthesis of the Hmd hydrogenase active site cofactor. J Bacteriol 2010; 192:595-598 doi: 10.1128/JB.01125-09
9. **Betz JN, Boswell NW, Fugate CJ, Holliday GL, Akiva E *et al***. [FeFe]-hydrogenase maturation: insights into the role HydE plays in dithiomethylamine biosynthesis. *Biochemistry* 2015; 54:1807-1818 doi: 10.1021/bi501205e
10. **Nicolet Y, Rubach JK, Posewitz MC, Amara P, Mathevon C *et al***. X-ray structure of the [FeFe]-hydrogenase maturase HydE from *Thermotoga maritima*. *J Biol Chem* 2008; 283:18861-18872 doi: 10.1074/jbc.M801161200
11. **Posewitz MC, King PW, Smolinski SL, Zhang L, Seibert M et al.** Discovery of two novel radical S-adenosylmethionine proteins required for the assembly of an active [Fe] hydrogenase. J Biol Chem 2004; 279:25711-25720 doi: 10.1074/jbc.M403206200

12. **Driesener RC, Duffus BR, Shepard EM, Bruzas IR, Duschene KS et al**. Biochemical and kinetic characterization of radical S-adenosyl-L-methionine enzyme HydG. Biochemistry 2013; 52:8696-8707 doi: 10.1021/bi401143s
13. **Leipe DD, Wolf YI, Koonin EV, Aravind L**. Classification and evolution of P-loop GTPases and related ATPases. *J Mol Biol* 2002; 317:41-72 doi: 10.1006/jmbi.2001.5378
14. **Brazzolotto X, Rubach JK, Gaillard J, Gambarelli S, Atta M *et al***. The [Fe-Fe]-hydrogenase maturation protein HydF from *Thermotoga maritima* is a GTPase with an iron-sulfur cluster. *J Biol Chem* 2006; 281:769-774 doi: 10.1074/jbc.M510310200
15. **Bortolus M, Costantini P, Doni D, Carbonera D.** Overview of the Maturation Machinery of the H-Cluster of [FeFe]-Hydrogenases with a Focus on HydF. Int J Mol Sci 2018; 19:3118 doi: 10.3390/ijms19103118
16. **Shepard EM, Byer AS, Betz JN, Peters JW, Broderick JB.** A Redox Active [2Fe-2S] Cluster on the Hydrogenase Maturase HydF. Biochemistry 2016; 55:3514-3527 doi: 10.1021/acs.biochem.6b00528
17. **Blokesch M, Rohrmoser M, Rode S, Böck A**. HybF, a zinc-containing protein involved in NiFe hydrogenase maturation. *J Bacteriol* 2004; 186:2603-2611 doi: 10.1128/JB.186.9.2603-2611.2004
18. **Miki K, Atomi H, Watanabe S.** Structural Insight into [NiFe] Hydrogenase Maturation by Transient Complexes between Hyp Proteins. Acc Chem Res 2020; 53:875-886 doi: 10.1021/acs.accounts.0c00022
19. **Watanabe S, Sasaki D, Tominaga T, Miki K**. Structural basis of [NiFe] hydrogenase maturation by Hyp proteins. Biol Chem 2012; 393:1089-1100 doi: 10.1515/hsz-2012-0197
20. **Sydor AM, Lebrette H, Ariyakumaran R, Cavazza C, Zamble DB**. Relationship between Ni(II) and Zn(II) coordination and nucleotide binding by the *Helicobacter pylori* [NiFe]-hydrogenase and urease maturation factor HypB. *J Biol Chem* 2014; 289:3828-3841 doi: 10.1074/jbc.M113.502781
21. **Thomas C, Waclawek M, Nutschan K, Pinske C, Sawers RG.** The Extended C-Terminal α-Helix of the HypC Chaperone Restricts Recognition of Large Subunit Precursors by the Hyp-Scaffold Machinery during [NiFe]-Hydrogenase Maturation in Escherichia coli. J Mol Microbiol Biotechnol 2018; 28:87-97 doi: 10.1159/000489929
22. **Edgar RC**. MUSCLE: multiple sequence alignment with high accuracy and high throughput. *Nucleic Acids Res* 2004; 32:1792-1797 doi: 10.1093/nar/gkh340
23. **Yang J, Yan R, Roy A, Xu D, Poisson J *et al***. The I-TASSER Suite: protein structure and function prediction. *Nat Methods* 2015; 12:7-8 doi: 10.1038/nmeth.3213
24. **Roy A, Kucukural A, Zhang Y**. I-TASSER: a unified platform for automated protein structure and function prediction. *Nat Protoc* 2010; 5:725-738 doi: 10.1038/nprot.2010.5

25. **Zhang Y**. I-TASSER server for protein 3D structure prediction. *BMC Bioinformatics* 2008; 9:40 doi: 10.1186/1471-2105-9-40

26. **Yu NY, Wagner JR, Laird MR, Melli G, Rey S *et al***. PSORTb 3.0: improved protein subcellular localization prediction with refined localization subcategories and predictive capabilities for all prokaryotes. *Bioinformatics* 2010; 26:1608-1615 doi: 10.1093/bioinformatics/btq249

27. **de Castro E, Sigrist CJ, Gattiker A, Bulliard V, Langendijk-Genevaux PS *et al***. ScanProsite: detection of PROSITE signature matches and ProRule-associated functional and structural residues in proteins. *Nucleic Acids Res* 2006; 34:W362-365 doi: 10.1093/nar/gkl124

28. **Bendtsen JD, Nielsen H, Widdick D, Palmer T, Brunak S**. Prediction of twin-arginine signal peptides. *BMC Bioinformatics* 2005; 6:167 doi: 10.1186/1471-2105-6-167

29. **Sanders C, Turkarslan S, Lee DW, Daldal F**. Cytochrome c biogenesis: the Ccm system. *Trends Microbiol* 2010; 18:266-274 doi: 10.1016/j.tim.2010.03.006

30. **Stevens JM, Mavridou DA, Hamer R, Kritsiligkou P, Goddard AD *et al***. Cytochrome c biogenesis System I. *FEBS J* 2011; 278:4170-4178 doi: 10.1111/j.1742-4658.2011.08376.x

31. **Kranz RG, Richard-Fogal C, Taylor JS, Frawley ER**. Cytochrome c biogenesis: mechanisms for covalent modifications and trafficking of heme and for heme-iron redox control. *Microbiol Mol Biol Rev* 2009; 73:510-528 doi: 10.1128/MMBR.00001-09

32. **Layer G**. Heme biosynthesis in prokaryotes. *Biochim Biophys Acta Mol Cell Res* 2021; 1868:118861 doi: 10.1016/j.bbamcr.2020.118861

33. **Weiner JH, Bilous PT, Shaw GM, Lubitz SP, Frost L *et al***. A Novel and Ubiquitous System for Membrane Targeting and Secretion of Cofactor-Containing Proteins. *Cell* 1998; 93:93-101 doi: 10.1016/S0092-8674(00)81149-6

34. **Sargent F, Bogsch EG, Stanley NR, Wexler M, Robinson C *et al***. Overlapping functions of components of a bacterial Sec-independent protein export pathway. *EMBO J* 1998;17:3640-3650 doi: 10.1093/emboj/17.13.3640

35. **Wu LF, Chanal A, Rodrigue A**. Membrane targeting and translocation of bacterial hydrogenases. *Arch Microbiol* 2000;173:319-324 doi: 10.1007/s002030000144

36. **Gross R, Simon J, Kroger A**. The role of the twin-arginine motif in the signal peptide encoded by the hydA gene of the hydrogenase from wolinella succinogenes. Arch Microbiol 1999; 172:227-232 doi: 10.1007/s002030050764

37. **Ignatova Z, Hörnle C, Nurk A, Kasche V**. Unusual signal peptide directs penicillin amidase from *Escherichia coli* to the Tat translocation machinery. *Biochem Biophys Res Commun* 2002; 291:146-149 doi: 10.1006/bbrc.2002.6420

38. **Stanley NR, Palmer T, Berks BC**. The twin arginine consensus motif of Tat signal peptides is involved in Sec-independent protein targeting in *Escherichia coli*. *J Biol Chem* 2000; 275:11591-11596 doi: 10.1074/jbc.275.16.11591

39. **Ize B, Gérard F, Zhang M, Chanal A, Voulhoux R *et al***. In vivo dissection of the Tat translocation pathway in *Escherichia coli*. *J Mol Biol* 2002; 317:327-335 doi: 10.1006/jmbi.2002.5431

40. **DeLisa MP, Samuelson P, Palmer T, Georgiou G.** Genetic analysis of the twin arginine translocator secretion pathway in bacteria. J Biol Chem 2002; 277:29825-29831 doi: 10.1074/jbc.M201956200

41. Prabudiansyah I, Driessen AJM. The Canonical and Accessory Sec System of Gram-positive Bacteria. *Curr Top Microbiol Immunol* 2017; 404:45-47 doi: 10.1007/82_2016_9

42. **Smets D, Loos MS, Karamanou S, Economou A**. Protein Transport Across the Bacterial Plasma Membrane by the Sec Pathway. *Protein J* 2019; 38:262-273 doi: 10.1007/s10930-019-09841-8

43. **Köpke M, Held C, Hujer S, Liesegang H, Wiezer A *et al***. *Clostridium ljungdahlii* represents a microbial production platform based on syngas. *Proc Natl Acad Sci U S A* 2010; 107:13087-13092 doi: 10.1073/pnas.1004716107

44. **Nagarajan H, Sahin M, Nogales J, Latif H, Lovley DR *et al***. Characterizing acetogenic metabolism using a genome-scale metabolic reconstruction of *Clostridium ljungdahlii*. *Microb Cell Fact* 2013; 12:118 doi: 10.1186/1475-2859-12-118

45. **Lee J, Lee JW, Chae CG, Kwon SJ, Kim YJ *et al***. Domestication of the novel alcohologenic acetogen *Clostridium* sp. AWRP: from isolation to characterization for syngas fermentation. *Biotechnol Biofuels* 2019; 12:228 doi: 10.1186/s13068-019-1570-0

46. **Song Y, Lee JS, Shin J, Lee GM, Jin S *et al***. Functional cooperation of the glycine synthase-reductase and Wood-Ljungdahl pathways for autotrophic growth of *Clostridium drakei*. *Proc Natl Acad Sci U S A* 2020; 117:7516-7523 doi: 10.1073/pnas.1912289117

47. **Mulder DW, Boyd ES, Sarma R, Lange RK, Endrizzi JA *et al***. Stepwise [FeFe]-hydrogenase H-cluster assembly revealed in the structure of HydA(DeltaEFG). *Nature* 2010; 465:248-251 doi: 10.1038/nature08993

48 **Britt RD, Rao G, Tao L**. Biosynthesis of the catalytic H-cluster of [FeFe] hydrogenase: the roles of the Fe-S maturase proteins HydE, HydF, and HydG. *Chem Sci* 2020; 11:10313-10323 doi: 10.1039/d0sc04216a

49. **Mehta N, Olson JW, Maier RJ**. Characterization of *Helicobacter pylori* nickel metabolism accessory proteins needed for maturation of both urease and hydrogenase. *J Bacteriol* 2003; 185: 726-734 doi: 10.1128/JB.185.3.726-734.2003

50. **Lacasse MJ, Summers KL, Khorasani-Motlagh M, George GN, Zamble DB**. Bimodal Nickel-Binding Site on *Escherichia coli* [NiFe]-Hydrogenase Metallochaperone HypA. *Inorg Chem* 2019; 58:13604-13618 doi: 10.1021/acs.inorgchem.9b00897

51. **Watanabe S, Arai T, Matsumi R, Atomi H, Imanaka T *et al***. Crystal structure of HypA, a nickel-binding metallochaperone for [NiFe] hydrogenase maturation. *J Mol Biol* 2009; 394:448-459 doi: 10.1016/j.jmb.2009.09.030

52. **Xia W, Li H, Yang X, Wong KB, Sun H**. Metallo-GTPase HypB from *Helicobacter pylori* and its interaction with nickel chaperone protein HypA. *J Biol Chem* 2012; 287:6753-6763 doi: 10.1074/jbc.M111.287581

53. **Sasaki D, Watanabe S, Matsumi R, Shoji T, Yasukochi A *et al***. Identification and structure of a novel archaeal HypB for [NiFe] hydrogenase maturation. J Mol Biol. 2013 May 27;425(10):1627-40. doi: 10.1016/j.jmb.2013.02.004

54. **Blokesch M, Böck A**. Properties of the [NiFe]-hydrogenase maturation protein HypD. *FEBS Lett* 2006; 580:4065-4068 doi: 10.1016/j.febslet.2006.06.045
